# Penile calciphylaxis in a patient with end-stage renal disease and chronic hemodialysis: a case report

**DOI:** 10.11604/pamj.2022.43.136.37673

**Published:** 2022-11-14

**Authors:** Saad Bkiri, Zakaria Tlemsani, Youness Khdach, Youssef Nmili, Karim Bennani, Fayçal Abbad, Mohammed Ghadouane

**Affiliations:** 1 Urology Department, Cheikh Zaid International Hospital, Abulcasis International University, Rabat, Morocco,; 2 Nephrology Department, Cheikh Zaid International Hospital, Abulcasis International University, Rabat, Morocco,; 3 Histopathology Department, Cheikh Zaid international Hospital, Abulcasis International University, Rabat, Morocco

**Keywords:** Penile calciphylaxis, renal failure, total penectomy, case report

## Abstract

Penile calciphylaxis is a rare and highly morbid condition mainly affecting diabetic patients with chronic renal failure (CRF). It is characterized by ischemic skin ulceration and necrosis secondary to dystrophic calcification of the subcutaneous penile tissue and penile arterioles. We report a 52-year-old male with a 6-year history of diabetes mellitus and CRF on hemodialysis, who presented with a painful penile necrotic lesion in the last three weeks. He firstly treated with medical treatment, which was failed. Then underwent total penectomy. The histopathology result confirmed the diagnosis of penile calciphylaxis. Unfortunately, he passed away due to septic shock and multisystem organ failure ten days after surgery. In conclusion, the diagnosis of penile calciphylaxis must be evoked in the presence of any minimal necrotic penile lesion in a patient with CRF; this will initiate quick medical and/or minimally invasive surgical treatment to improve the patient's prognosis and avoid serious complications.

## Introduction

Calciphylaxis, or calcifying uremic arteriopathy, is a very rare and severely morbid condition [1,2]. This disorder is characterized by the deposition of calcium in the small arterial vessels of the dermis and subdermal adipose tissue, leading first to their ischemia, then to their occlusion, and finally to necrosis and gangrene [3]. This disease is generally associated with high circulating calcium and phosphate levels. However, several cases have been reported with normal serum levels [3].

Penile calciphylaxis is a severe condition rarely reported and associated with a poor prognosis [4]. Until today the management of penile calciphylaxis still represents a real challenge for the medical community since there is no well-established protocol [1,4]. We report a case of a diabetic mellitus and chronic hemodialysis patient presenting to our department with a necrotic painful penile lesion.

## Patient and observation

**Figure 1:**
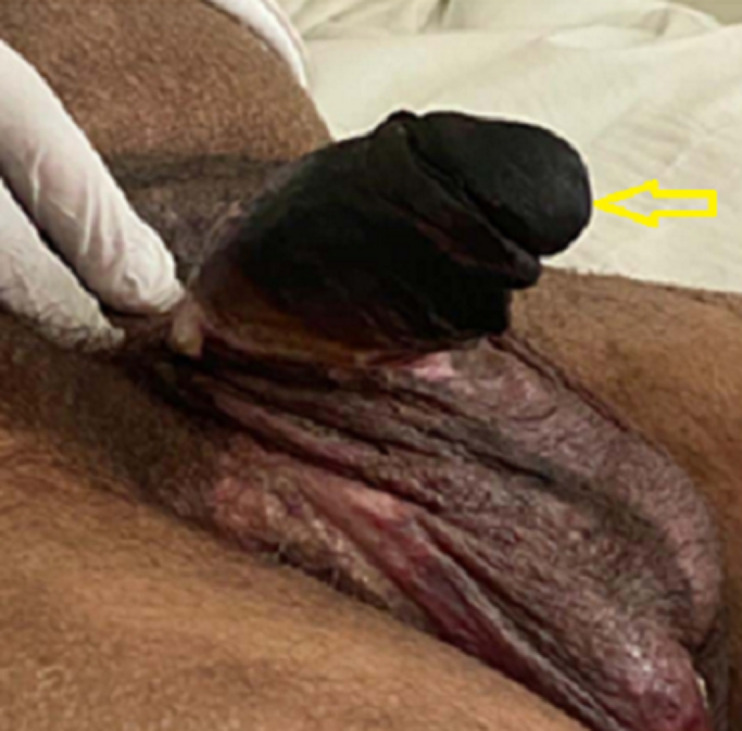
penile necrosis (arrow)

**Patient information:** a 52-year-old male presented to our hospital for the gradual development (for three weeks) of a very painful necrotic lesion involving the whole penis (Figure 1). The patient has diabetes mellitus and chronic renal failure (CRF), undergoing hemodialysis for 6 years. He also suffered from diabetic retinopathy and Charcot neuro-osteoarthropathy in the left foot. In 2015, he was diagnosed with ankylosing spondylitis and is currently receiving tumor necrosis factor (TNF)-alpha inhibitors.

**Figure 2:**
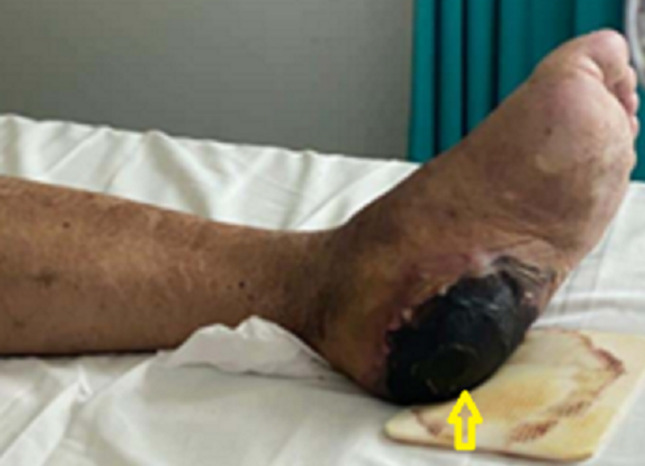
bedsore of the left heel with Charcot’s neuro-osteoarthropathy (arrow)

**Clinical finding:** the general examination revealed a febrile (temperature: 38.5°C), asthenic, anxious patient with necrotic bedsore on the left heel (Figure 2). Physical examination of the genitals confirmed a painful necrotic lesion engaging that entire penis associated with crepitations and a foul smell. No other perineo-scrotal lesions were observed.

**Diagnostic assessment:** the laboratory investigation showed leukocytosis with a neutrophil predominance (WBC: 15800/mm^3^, with neutrophile: 12343/mm^3^), thrombocytosis: 598000 cells//mm^3^, C-reactive protein (CRP): 20.6 mg/dL, parathormone (PTH): 3000pg/mL, serum phosphate: 7.3 mg/dL, and serum calcium: 10.4 mg/dL. The blood creatinine level was 12 mg/dl, potassium level was 5.8 mmol/L, blood sugar was 164 mg/dl, serum bicarbonate (HCO_3-_) level was 9mmol/l, and hemoglobin A1C (HbA1C) was 8.5%. The urine culture revealed coagulase-positive staphylococcus sensitive to ceftriaxone and vancomycin.

Neck ultrasonography (US) showed two left inferior parathyroid nodules measuring 16 mm and 18 mm, suggesting a secondary hyperparathyroidism diagnosis. Penile color Doppler US showed severe arteriosclerotic changes with stenosis and calcifications of the penile artery. After the presentation of this case in the multidisciplinary meeting and given this clinical and biological data, the diagnosis of penile calciphylaxis was retained. The parathyroidectomy was planned to schedule after the total penectomy.

**Figure 3:**
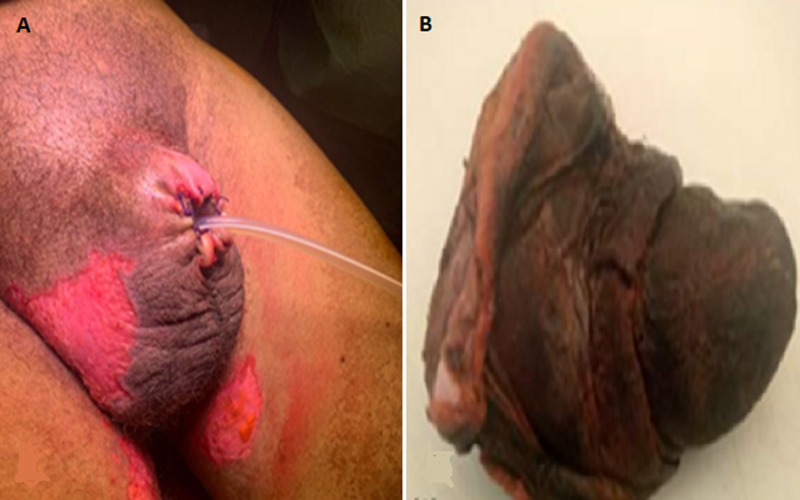
A) postoperative total penectomy; B) macroscopic aspect of the surgical specimen after formalin fixation

**Therapeutic intervention:** the patient was admitted to urology department, receiving parenteral antibiotics (ceftriaxone 1g every 12 hours and metronidazole 500mg every 24 hours), an alkalinization with bicarbonate serum, and correcting his blood sugar with regular insulin. Regarding secondary hyperparathyroidism, a treatment based on cinacalcet 30 mg 1 pill 2 times per day, phosphate blockers 800 mg 2 pill 3 times per day, and oral vitamin D 100,000 IU/15 days was conducted. Neuropathic pain was treated by pregabalin 25 mg one pill/day, tramadol 50mg one pill/day, and benfotiamine (vit B1) 100mg one pill 3 times per day. The patient was admitted to the operating room after taking two sessions of acute hemodialysis (48 hours between the two sessions). He received a daily subcutaneous injection of enoxaparin sodium (0.4 mg) and a daily omeprazole (20 mg) pill to prevent thrombo-embolic complications or stress ulcers. After a successful hemodynamic stabilization, the patient underwent surgical exploration. Intraoperatively, the total penile shaft was found to be completely necrotic, with greater than 75% of the penile corpora filled with pus, for which a total penectomy and excision of the left heel necrotic bedsore were performed (Figure 3).

**Figure 4:**
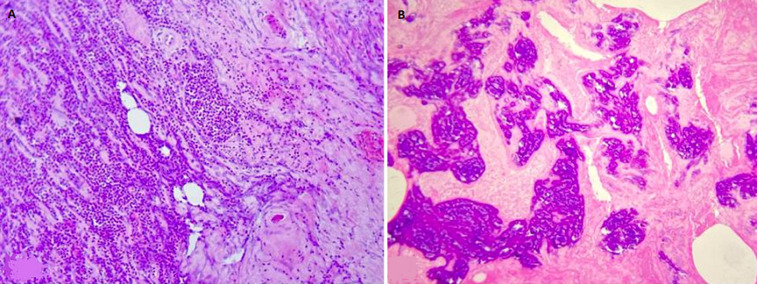
histological aspect of the excised part: A) micrograph (Gx20 - HE staining) showing the penile parenchyma totally remodeled by suppurative necrosis sites; B): micrograph (Gx20 - HE staining) showing extensive calcic deposits within totally ischemic and phantom areas

**Follow-up and outcome:** the patient developed a septic shock (blood pressure 61/40 mmHg) postoperatively. He was immediately admitted to the intensive care unit, receiving a fluid resuscitation and vasopressor drug agent (norepinephrine). The blood culture showed coagulase-positive staphylococcus sensitive only to ceftriaxone and vancomycin. The patient received parenteral vancomycin with a loading dose of 1 g and 500 mg as a maintenance dose. However, the patient's general condition worsened and passed away after ten days due to septic shock and multisystem organ failure. The histopathological examination revealed numerous foci of microcalcifications as well as extensive ischemic and necrotic remodeling on the entire penile parenchyma consistent with calciphylaxis (Figure 4).

**Patient perspective:** the patient was very positive about the efforts made by all the medical and paramedical staff during his hospitalization in the urology department. The patient's family understood the gravity of the illness.

**Informed consent:** written informed consent was obtained from the patient family.

## Discussion

Calciphylaxis, also known as calcific uremic arteriopathy, is a sporadic but fatal vascular disease [5]. It is characterized by calcifications of the blood vessels in the dermis and subdermal fatty tissue, leading to ischemia and skin necrosis [2].

On the epidemiological aspect, calciphylaxis has an estimated annual incidence of 35 cases per 10,000 hemodialysis patients in the United States [6]. This high occurrence of calciphylaxis can be explained by the natural increase of this disease in chronic hemodialysis patients, as well as the improved understanding and diagnosis by physicians [6]. Calciphylaxis incidence is estimated to be 1-4% in chronic hemodialysis patients [7]. It generally affects patients between 40 and 60 years old with a medical history of diabetes, atherosclerosis, end-stage renal disease (ESRD), and obesity [7]. Our patient fits in this spectrum.

The most common sites of skin necrosis are the buttocks, thighs, and abdomen [8]. Our patient developed penile necrosis. The penis is vascularized by three different arteries: dorsal, deep, and urethral arteries. This important vessel system makes the occurrence of necrosis in this region rare and gradual [8].

A biopsy can help make the diagnosis in doubtful cases. However, the risk of inducing wet gangrene and infection is very high [9]. Radiological tests may help to guide the diagnosis. Doppler US will allow studying of the vascularization of the penis, a computed tomography (CT) scan will objectify the vascular calcifications, and magnetic resonance imaging (MRI) will demarcate the extent of the necrotic lesion [7]. In our case, the diagnosis of penile calciphylaxis was based on clinical and biological findings; neither biopsy nor imaging was performed. The histopathology finding confirmed our diagnosis.

There is no consensus regarding a proven treatment of penile calciphylaxis. For both non-penile cutaneous calcific uremic arteriolopathy and non-cutaneous calcific uremic arteriolopathy, the widely used treatment is the conservative management of penile calciphylaxis mirrors. The management of less severe cases of penile calciphylaxis consists of local wound debridement, analgesia, and normalization of metabolic dysregulation [10,11]. In the presence of secondary hyperparathyroidism associated with hypercalcemia and/or hyperphosphatemia, medical treatment is based on phosphorus binders (sevelamer) and a calcimimetic agent (cinacalcet) [11,12]. Sodium thiosulfate is also widely used to treat calciphylaxis: it can be an alternative to penectomy in very minimal lesions [13]. Nonetheless, partial or total penectomy is necessary for the presence of infection and gangrene signs [7]. Parathyroidectomy was also a possible treatment option to restore phosphocalcique control. However, a recent study found no correlation between increased survival and parathyroidectomy [14].

Other reported treatment modalities for penile calciphylaxis include revascularization surgery, internal iliac artery stent, and hyperbaric oxygen therapy [1]. Akai *et al*. reported a case of penile calciphylaxis who underwent femoral artery to deep dorsal penile vein bypass with a successful outcome [15]. In Oikawa *et al*. case, the patient was treated with hyperbaric oxygen therapy, but he died within a month [16]. A multi-disciplinary approach is necessary to manage this condition, as it should involve a team of nephrologists, urologists, reconstructive surgeons, and palliative caregivers [11]. In our patient, the decision to intervene surgically was based on pain severity, fluctuance of the penile shaft, and extent of penile necrosis. The severity and extent of his disease obviated the possibility for conservative management, and intraoperatively, the finding of pus-filled corpora confirmed the need to proceed with total penectomy. A similar case with a total penectomy procedure was reported by Yang *et al*. [1].

The prognosis of penile calciphylaxis is very poor, with a very high risk of mortality estimated at 69% with an average time to death of 2.5 months after the diagnosis. The most common causes of death were septic shock, multisystem organ failure, stroke, myocardial infarction, pneumonia, and renal failure [17]. Our patient died ten days after a massive septic shock and multisystem organ failure.

## Conclusion

Penile calciphylaxis is a very rare condition with a poor prognosis. This condition should be evoked in every minimal necrotic lesion in the diabetic patient with ESRD; this will initiate quick medical and/or minimally invasive surgical treatment to improve the patient's prognosis and avoid serious complications.
